# Interactive digital tools to support empowerment of people with cancer: a systematic literature review

**DOI:** 10.1007/s00520-024-08545-9

**Published:** 2024-05-31

**Authors:** Leena Tuominen, Helena Leino-Kilpi, Jenna Poraharju, Daniela Cabutto, Carme Carrion, Leeni Lehtiö, Sónia Moretó, Minna Stolt, Virpi Sulosaari, Heli Virtanen

**Affiliations:** 1 Department of Nursing Science, University of Turku, Turku, Finland; 2 University of Turku FI and Wellbeing Services County of Southwest Finland, University of Turku FI, Turku University Hospital, Turku, Finland; 3 eHealth Lab Research Group, School of Health Sciences and eHealth Center, Universitat Oberta de Catalunya, Barcelona, Spain; 4 eHealth Lab Research Group, Faculty of Health Sciences Studies, E-Health Center, School of Health Sciences and eHealth Center, Universitat Oberta de Catalunya, Barcelona, Spain; 5 Turku University Library, University of Turku, Turku, Finland; 6 Wellbeing Services County of Satakunta, Pori, Finland; 7 Health and Well-Being, Turku University of Applied Sciences, Turku, Finland; 8 Research Advancing Supportive Cancer and Palliative care (CARE) - research group, Turku, Finland; 9 European Oncology Nursing Society, Brussels, Belgium

**Keywords:** Cancer care, Empowerment, Interactive digital tool, Oncology, People with cancer

## Abstract

**Purpose:**

To identify and synthesise interactive digital tools used to support the empowerment of people with cancer and the outcomes of these tools.

**Methods:**

A systematic literature review was conducted using PubMed, CINAHL, Web of Science, Cochrane, Eric, Scopus, and PsycINFO databases in May 2023. Inclusion criteria were patient empowerment as an outcome supported by interactive digital tools expressed in study goal, methods or results, peer-reviewed studies published since 2010 in cancer care. Narrative synthesis was applied, and the quality of the studies was assessed following Joanna Briggs Institute checklists.

**Results:**

Out of 1571 records screened, 39 studies published in 2011–2022 with RCT (17), single-arm trial (15), quasi-experimental (1), and qualitative designs (6) were included. A total of 30 interactive digital tools were identified to support empowerment (4) and related aspects, such as self-management (2), coping (4), patient activation (9), and self-efficacy (19). Significant positive effects were found on empowerment (1), self-management (1), coping (1), patient activation (2), and self-efficacy (10)*.* Patient experiences were positive. Interactivity occurred with the tool itself (22), peers (7), or nurses (7), physicians (2), psychologists, (2) or social workers (1).

**Conclusion:**

Interactive digital tools have been developed extensively in recent years, varying in terms of content and methodology, favouring feasibility and pilot designs. In all of the tools, people with cancer are either active or recipients of information. The research evidence indicates positive outcomes for patient empowerment through interactive digital tools. Thus, even though promising, there still is need for further testing of the tools.

**Supplementary Information:**

The online version contains supplementary material available at 10.1007/s00520-024-08545-9.

## Introduction

The growing number of people with cancer calls for new solutions for care and treatment [1]. Globally, an estimated 28.4 million new patients are predicted to have cancer in 2040, compared to 19.3 million in 2020 [2]. The physical, emotional, and financial strain of illness on the patients themselves is significant. Therefore, patient empowerment should be considered and acknowledged in healthcare digitalisation. [3] In this review, the focus is on interactive digital tools (IDTs) in the context of patient empowerment.

Empowerment is a multidimensional concept. In this review, it is seen as patient capacities and behaviours, both comprising cognition. As capacities, empowerment includes perceived control over own health and healthcare, experience of being respected, self-efficacy, and health literacy [4]. As behaviours, empowerment includes participation, actions made for decision-making, and self-management [4]. In cancer care, the definition of empowerment varies. It has been described in relation to pain management with the concepts self-efficacy, active participation, increased abilities, and control of life [5]. Empowerment has been measured as an outcome in terms of knowledge [6, 7], self-efficacy [5], and coping [8]. The variation in definitions has led to the development of different tools to measure empowerment and its aspects; however, they may not fully capture the idea of empowerment as a whole [9, 10] or are not intended for the cancer care context [11]. Empowerment can be investigated as such, but also through its various aspects which can be regarded as sub dimensions of empowerment. In this review, we have used aspects of self-care, self-management, coping, control, action, patient activation, and self-efficacy in addition to the concept itself due to our emphasis on empowerment as an outcome [12].

Digital technologies can strengthen health service delivery [3] by improving its accessibility and by providing tailored interventions that provide support at times when it is most needed [13]. Previous systematic reviews have focused on digital tools such as smartphone applications [13–16], web-based communication platforms [17, 18], and artificial intelligence [19] adopted during the cancer continuum with various purposes and outcomes. Mobile-based applications supporting the empowerment of people with cancer have been designed to provide information about cancer and treatment [13, 15, 16], support self-management [13, 15, 16] and shared decision-making [19], monitor and promote health and wellbeing [13, 15, 19], enhance communication skills [15], raise awareness of the illness, and assist in early detection and prevention [16]. In some cases, the tools have provided social [13, 16] and peer support [13, 15] as well as relaxation techniques [15].

Web-based platforms have increased access to cancer screening, although diagnostic accuracy has decreased in some cases [19]. In addition, they have increased knowledge about the disease, assisted in decision-making regarding prostate cancer screening, and improved positive health behaviour such as physical activity and weight loss among cancer survivors [19]. Monitoring patient-reported outcomes using web- or mobile-based digital tools has reduced the number of emergency admissions and hospitalisations and thus, reduced costs as well [19]. In psychological care, chatbot-based platforms have improved adherence to treatment, and virtual reality platforms have reduced distress and fatigue after chemotherapy among people with breast cancer [19] Web-based communication platforms facilitating communication between patients and health care professionals (HCPs) have improved communication [17] and have had a positive effect on cancer-related symptoms [17, 18] and their reporting [17, 18], functional capacity [18], decision-making [17], health care utilisation, e.g., clinical visits and calls [17], and quality of life [17, 18] among people with cancer. A mobile-based self-management intervention has been effective for self-efficacy, self-management, exercise compliance, and quality of life, but not for symptom relief, role-functioning, depression, or social support among people with breast cancer [14]. Due to lack of reporting, no conclusions can be drawn about the mechanisms of digital tools that lead to change in outcomes.

Despite existing reviews, there is still a need for an extensive review of literature on the outcomes of IDTs to support patient empowerment in cancer care. In this review, interactivity refers to patients interacting with the tool itself, peers, voluntary sector actors or HCPs, indicating that the person is active, respected and aims to be empowered, supported by the tool. Interactive digital tools designed purely for peer support (e.g., social media and chat forums) were excluded as they were considered their own, separate area of interest.

The purpose of this systematic literature review is to identify and synthesise the IDTs used to support the empowerment of people with cancer and the outcomes of these tools. The research questions are as follows:

1. What interactive digital tools are used to support empowerment among people with cancer? and
2. What are the outcomes of these interactive digital tools used among people with cancer?

## Methods

This systematic review adheres to the Preferred Reporting Items for Systematic reviews and Meta-Analyses (PRISMA) [20].

### Literature search

In the review, studies published 01/2010–05/2023 were accepted, assuming the active development of digital tools during this time [21]. Inclusion and exclusion criteria are presented in Table 1.

**Table 1:** Eligibility criteria of included studies

Inclusion criteria	Exclusion criteria
Patient empowerment or related aspects (self-care, self-management, coping, control, action, activation, self-efficacy) is an outcome supported by interactive digital tool(s)	Patient empowerment or related aspects (self-care, self-management, coping, control, action, activation, self-efficacy) supported by interactive digital tools is not an outcome
Interactive digital tool(s) used by patients themselves or together with significant others, peers, voluntary sector actors, and different groups of HCPs	Chat forums or social media. Interactive digital tool(s) used by somebody else
Patient empowerment or related aspects (self-care, self-management, coping, control, action, activation, self-efficacy) supported by interactive digital tools expressed/described in the aim or methods or results of the study report (article/publication)	Patient empowerment or related aspects (self-care, self-management, coping, control, action, activation, self-efficacy) supported by interactive digital tools expressed/described in other parts than aim or methods or results of the study report (article/publication)
Peer-reviewed research papers with different designs	Protocol articles, reviews, posters, conference abstracts, proceedings, books/book chapters, editorials, letters, notes, data papers
Setting: oncology, cancer care, adults	Setting: other than oncology, cancer care, children
Published ≥ 2010	Published prior to 2010

A systematic literature search was conducted in collaboration with an Information Specialist using seven databases: PubMed, CINAHL, Web of Science, Scopus, Cochrane, PsycINFO, and ERIC with the following keywords: empowerment, cancer, digital, patient, and interactive. For the full search strategies, see Appendix 1 (supplement). Additionally, citation searching of the included articles was conducted. The search was limited to peer-reviewed research papers and English language. Covidence systematic review software [22] was used to manage the systematic review process. First, duplicates were removed. Next, two reviewers screened each report independently based on title and abstract against the inclusion and exclusion criteria. Finally, full texts were screened, and decision was made of studies to be included in the review. Conflicts were solved by a third reviewer. Data were extracted independently by two researchers, including information of authors, year, country, purpose, design and setting, theoretical approach, variables, participant characteristics, data collection and analysis method, description of interactive digital tool, and outcomes of the study in terms of patient empowerment or related aspects (self-care, self-management, coping, control, action, activation, or self-efficacy).

### Synthesis methods

Studies were grouped for the synthesis by (1) research design, (2) elements of the IDTs, i.e., activities addressed to patients or HCPs, and (3) outcomes of the tools on empowerment and related aspects. Two kinds of evidence were explored: statistical and experiential. Statistical evidence was used for the analysis of quantitative studies; synthesis is based on statistically significant differences between or within the groups using descriptive quantification and a narrative summary of the data (Table 2). Experiential evidence was used for the analysis of qualitative studies; synthesis is based on patients’ experiences related to empowerment when using IDTs. Narrative synthesis was used to integrate the evidence of the studies [23]. Conclusions were made based on either statistical or experiential scientific evidence of the IDTs to support empowerment of people with cancer. Explanations of the abbreviations of the IDTs are provided in Appendix 2 (supplement).

**Table 2:** Study characteristics (*n* = 39)

Reference Author(s) Year of publication Country Reference number	Purposes	Design	Participants	Intervention, interactive digital tool, length of use, interaction with patients	Data collection instruments of empowerment and related aspects	Statistically significant outcomes for empowerment and related aspects	Quality appraisal JBI
Absolom et al. 2021 UK [25]	To assess the effect of the mobile-based electronic patient self-Reporting of Adverse-events: Patient Information and aDvice (eRAPID) for symptom-reporting during cancer treatment	RCT	People with colorectal, breast, or gynaecological cancers during treatment (*n* = 508) Mean age 56 Female 79.9% Male 20.1%	IG: eRAPID CG: standard care 18 weeks Clinical team monitored by nurses	Self-Efficacy Scale for Managing Chronic Disease questionnaire CBI-B PAM	A statistically significant effect on self-efficacy (*p* = 0.007) in IG A statistically non-significant effect on patient activation	10/13
Beatty et al. 2011 Australia [26]	To assess the feasibility and efficacy of web-based Cancer Coping Online (CCO) for reducing cancer-related distress	A single-arm pilot feasibility study	People with breast, gynaecological, lymphoma or bowel cancer during treatment with curative intent (*n* = 12) Mean age 48.33 Female 91.7% Male 0.3%	CCO 6 weeks The tool	Mini-MAC	Reductions in three maladaptive coping styles with medium effect sizes: helplessness/hopelessness (*d* = 0.64); anxious/preoccupation (*d* = 0.43); and fatalism (*d* = 0.41). Statistical significance not reported	6/9
Beatty et al. 2016 Australia [27]	To assess the feasibility and effect of web-based Cancer Coping Online (CCO) for reducing cancer-related distress	RCT	People with cancer during treatment with curative intent (*n* = 60) Median age 50.5 Female 95% Male 5%	IG: CCO CG: the web-based attention control 6 weeks The tool	Mini-MAC	A statistically non-significant effect on coping	10/13
Bender et al. 2022 Canada [28]	To assess the feasibility and effect of mobile/web-based True North Peer Navigation (PN) to support the prostate cancer journey	Single-arm pilot feasibility study	People with prostate cancer during treatment or survivors (*n* = 29) Mean age 65.2 Female 14.7% Male 85.3%	True North PN app 3 months Peers	PAM	A statistically significant effect on patient activation: baseline scores 62.3 (SD 20.9) post intervention scores 74.06 (SD 16.45), *p* < 0.01	6/9
Børøsund et al. 2014 Norway [29]	To compare effects of the web-based patient provider communication service (IPPC) and the illness management support program, WebChoice	RCT	People with breast cancer during treatment (*n* = 167) IG: median age 51 Female 100% IG2: median age 50 Female 100% GC: median age 53 Female 100%	IG: WebChoice group (IPPC included) IG 2: IPPC GC: standard care Preliminary findings from 6 months’ follow-up data in a 12-month trial Peers, nurses, physicians and social workers	CBI version 2.0	A statistically non-significant effect on self-efficacy	10/13
Bouma et al. 2017 Netherlands [30]	To examine the feasibility and effect of web-based information and support system	RCT feasibility study	Newly diagnosed people with neuroendocrine tumour during treatment (*n* = 20) GC: median age 64 Female 50% Male 50% IG: median age 59.5 Female 60% Male 40%	IG: web-based information and support system GC: standard care 12 weeks HCPs (professionals not reported in detail)	Dutch Constructs Empowering Outcomes questionnaire Semi-structured interviews	In IG, a positive moderate effect on empowerment in terms of being better informed (effect size 0.51) compared to GC. Statistical significance not reported	10/13
Cockle-Hearne et al. 2018 UK [31]	To assess the feasibility and effect of web-based getting down to coping to reduce distress after prostate cancer treatment	A single-arm mixed methods study	People with mild and moderate distress after treatment for prostate cancer (*n* = 30) Phase I, mean age 69 Phase II, mean age 64 Male 100%	Getting down to coping 4 weeks Peers, psychological practitioners, nurses	Self-Efficacy For Symptom Control Inventory	A statistically significant effect on self-efficacy (*p* = 0.02, *r* = − 0.412) A non-significant effect on managing symptoms or in performing daily activities	7/9
De Veer et al. 2020 Netherlands [32]	To assess the effect of a mobile-based self-management support intervention with an integrated eHealth application, Oncokompas	A single-arm mixed-methods study	People with cancer receiving support in the home setting in palliative care or survivors (*n* = 36) Age: 40% were 61–70 years Female 50% Male 50%	Oncokompas 12 weeks The tool	PAM	A statistically non-significant effect on patient activation	6/9
Dorfman et al. 2019 US [33]	To assess feasibility and effect of web-based pain coping skills training protocol, mPCST-community	A single-arm pilot study	People with breast cancer and pain from medically underserved areas during treatment or survivors (*n* = 20) Mean age 57.85 Female 100%	mPCST-community 5 weeks Psychologists	The self-efficacy for pain management subscale of the Chronic Pain Self-Efficacy Scale	A statistically significant effect on self-efficacy for pain management:* t* = 3.57, *p* = 0.0004, 95% CI [9.01, 30.90]	6/9
Fu et al. 2016 US [34]	To test web-based Optimal-Lymph-Flow (TOLF) system focusing on self-assessment and self-care strategies of lymphedema	A single-arm pilot study	People with surgical treatment with or without being diagnosed or treated for lymphedema (*n* = 20) Mean age 55.9 Female 100%	TOLF 12-week The tool	Patients’ report of self-care behaviours using self-care behaviour checklist hosted by TOLF	In IG, a statistically significant effect on self-management: less pain (*p* = 0.031), soreness (*p* = 0.021), aching *(p* = 0.024), tenderness (*p* = 0.039), fewer numbers of lymphoedema symptoms (*p* = 0.003), and improved symptom distress (*p* = 0.000) compared to CG	6/9
Groen et al. 2017 Netherlands [35]	To assess the feasibility and effect of a mobile-based supportive portal, MyAVL	A single-arm pilot feasibility study	People with lung cancer currently or recently treated with curative intent (*n* = 37) Mean age 59.6 years Female 47% Male 53%	MyAVL 4 months The tool	PAM	A statistically non-significant effect on patient activation	6/9
Kuijpers et al. 2016 Netherlands [36]	To assess the feasibility and effect of a mobile-based supportive portal, MijnAVL	A single-arm pilot feasibility study	Cancer survivors currently or recently treated with curative intent (*n* = 92) Mean age 49.5 Female 100%	MijnAVL 4 months The tool	PAM	A statistically non-significant effect on patient activation	6/7
Leach et al. 2022 US [37]	To assess the effect of a web-based self-management program, Springboard Beyond Cancer (SBC)	RCT	People with history of cancer during diagnosis, treatment or follow-up (*n* = 176) Age NR IG: female 92% Male 8% CG: female 88.6% Male 10.2%	IG: SBC and self-management text message program CG: access to a website built for the study 4 weeks The tool	the Stanford Self-Efficacy for Managing Chronic Disease Scale	A statistically significant effect on self-efficacy for managing cancer (*p* = 0.02)	9/13
Lee et al. 2014 South Korea [38]	To assess the feasibility and effect of a web-based self-management exercise and diet intervention (WSEDI)	RCT pilot feasibility study	Breast cancer survivors (*n* = 57) IG: mean age 43.2 Female 100% CG: mean age 41.5 Female 100%	WSEDI 12 weeks The tool	Self-efficacy (participants were asked, in terms of each goal behaviour, “How sure are you…)	A statistically significant effect on self-efficacy for exercise management (*p* = 0.024) and fruit and vegetables’ intake (*p* = 0.023)	11/13
Ma et al. 2021 US [39]	To assess the effect of a web-based automated chatbot, The Northwell Head & Neck Health Chats, for symptom self-management	A single-arm mixed method study	People with head and neck cancer during radiotherapy treatment (*n* = 84) Mean age 61.3 Female 28.6% Male 71.4%	The Northwell Head & Neck Health Chats 4 months Nurses, physicians, advanced care practitioners	Patient-reported outcomes	Of patients who had used chats, 61% reported that it helped with symptom self-management and reduced the need to call the care team. Statistical significance not reported	6/9
Maguire et al. 2021 Austria, Greece, Norway, Ireland, and UK [40]	To evaluate the effect of a mobile-based advanced symptom management system (ASyMS) to remote monitoring of chemotherapy-related side-effects	RCT	People with non-metastatic breast cancer, colorectal cancer, Hodgkin’s disease or non-Hodgkin’s lymphoma receiving first chemotherapy (*n* = 829) Mean age 52.4 Female 81.8% Male 18.2%	IG: ASyMS CG: standard care Over 6 cycles of chemotherapy Clinicians (professional groups not reported)	CASE-cancer	A statistically significant effect on self-efficacy (mean difference 0.81, 0.19 to 1.43; *p* = 0.01) in IG compared to CG	11/13
Maguire et al. 2015 UK [41]	To explore the feasibility and effect of mobile-based advanced symptom management system for radiotherapy (ASyMS-R)	A single-arm mixed-methods study	People with lung cancer receiving radiotherapy (*n* = 16) Mean age 63.6 Female 68.8% Male 31.2%	ASyMS-R Radiation therapy treatment + 1 month Clinicians (professional groups not reported)	SUPPH-29	A statistically non-significant effect on self-efficacy	6/9
Manne et al. 2020 US [42]	To evaluate the feasibility and effect of a web-based B-Sure to facilitate informed decisions	RCT pilot feasibility study	People with unilateral, nonhereditary breast cancer in the early treatment considering contralateral prophylactic mastectomy (*n* = 93) IG: mean age 47.5 Female 100% GC: mean age 45.5 Female 100%	IG: B-sure CG: standard care 4 weeks The tool	Self-efficacy (3-item measure)	A statistically non-significant effect on self-efficacy to manage worries	8/13
Melissant et al. 2018 Netherlands [43]	To assess the feasibility and effect of a mobile-based self-management support intervention with an integrated eHealth application, Oncokompas (including a breast cancer module)	A single-arm feasibility study	Breast cancer survivors (*n* = 101) Mean age 56 Female 100%	Oncokompas – and the breast cancer module 1 week The tool	PAM	A statistically significant effect on patient activation (mean 60.5 vs. 55.8) with a small effect size (*p* = 0.007, *r* = 0.24)	6/9
Murphy et al. 2022 US [44]	To assess the feasibility and effect of a mobile-based iManage-PC, a symptom monitoring and self-management program	A single-arm pilot study	People with prostate cancer during treatment (*n* = 96) Mean age 64.5 Male 100%	iManage-PC 4 weeks Peers	The Self-Efficacy for Managing PC Symptoms and Side Effects tool	A statistically significant effect on self-efficacy to manage adverse effects with medium effect size (*p* < 0.001, *r* = 0.39)	6/9
Northouse et al. 2014 US [45]	To assess the feasibility and effect of a web-based tailored psychoeducational FOCUS program for cancer patients and their family caregivers	A single-arm feasibility study	People with lung, breast, colorectal, or prostate cancer during cancer trajectory (*n* = 38) Mean age 54.8 Female 57.9% Male 42.1%	FOCUS Program 8 weeks The tool	Coping: Lewis Mutuality and Interpersonal Sensitivity Scale Brief version of the Social Support Scale Lewis Cancer Self-Efficacy Scale	A statistically non-significant effect on coping	6/9
Peipert et al. 2021 US [46]	To test the effect of a web-based CancerHelp-TT, a multimedia patient education software	RCT	People with stage I–III breast or colorectal cancer during treatment (*n* = 129) IG: mean age 52.6 Female 83.1% Male 16.9% CG: mean age 51.1 Female 82.8% Male 17.2%	IG: CancerHelp-TT CG: standard care (cancer education) Average 8 months The tool	CASE-cancer	A statistically non-significant effect on self-efficacy	9/13
Petrocchi et al. 2021 Switzerland and Italy [47]	To test the feasibility and effect of a mobile**-**based CSSI app to navigate the breast cancer journey	A single-arm mixed method study	People with breast cancer during treatment (*n* = 20) Mean age 51 Female 100%	CSSI app At least 1 month The tool	The Empower-ment Scale	A statistically significant effect on patient empowerment (*B* = 0.31, 95% CI 0.22–0.69; *p* = 0.009)	7/8
Poort et al. 2021 US [48]	To assess the feasibility and effect of a web-based IAYA for coping with cancer as a young adult	A single-arm pilot feasibility study	People with cancer during treatment (*n* = 25) Mean age 28 Female 56% Male 44%	IAYA 12 weeks Peers	1) CBI-B 2) PROMIS (self-efficacy for managing emotions and perceived emotional support)	A statistically non-significant effect on self-efficacy	6/9
Ruland et al. 2013 Norway [49]	To assess the effect of a web-based illness management support program, WebChoice	RCT	People with breast or prostate cancer during treatment (*n* = 325) IG: mean age 56.9 60% Female 40% Male CG: mean age 56.3 57% Female 43% Male	IG: WebChoice CG: URLs of publicly available cancer web sites 12 months Peers, nurses	The Cancer Behaviour Inventory version 2.0	A statistically non-significant effect on self-efficacy	10/13
Schuit et al. 2022 Netherlands [50]	To assess the effect of a mobile-based self-management support intervention with an integrated eHealth application, Oncokompas	RCT	Incurably ill people with cancer with a life expectancy of more than 3 months (*n* = 138) Mean age 61.1 Female 46% Male 54%	IG: Oncokompas CG: standard care 3 months The tool	PAM, GSE	A statistically non-significant effect on patient activation and self-efficacy	9/13
Tagai et al. 2021 US [51]	To assess the effect of a web-based PROGRESS program for adaptive coping among cancer survivors	RCT	People with localised prostate cancer within 1 year of treatment completion (*n* = 431) IG: mean age 63.8 GC: mean age 63.2 Male 100%	IG: PROGRESS GC: enhanced standard care 6 months The tool	CCQ, the self‐efficacy for re‐entry scale, self‐efficacy for symptom control scale	A statistically significant effect on coping, i.e., healthy redirection of worrying thoughts about cancer (*F* = 7.914, *p* < 0.01) and on decrease in interpersonal coping in IG compared to CG (*F* = 6.201, *p* < 0.05). Positive coping decreased statistically significantly in both groups over time (*F* = 11.613, *p* < 0.001) A statistically non-significant effect on self‐efficacy for symptom control	9/13
van Bruinessen et al. 2016 Netherlands [52]	To assess the effect of a web-based communication tool, PatientTIME	RCT	People with malignant lymphoma during treatment or survivors (*n* = 87) Mean age 56 Female 61% Male 39%	IG 1: PatientTIME IG 2: PatientTIME and audio GC: standard care Length NR The tool	Perceived Efficacy in Patient-Physician Interactions (PEPPI)	A statistically significant effect on self-efficacy (− 1.97 points, *p* = 0.02) in IG compared to CG	9/13
van den Berg et al. 2015 Netherlands [53]	To assess the effect of a web-based self-help program for psychological adjustment after primary breast cancer, BREATH	RCT	Breast cancer survivors (*n* = 150) IG: mean age 51.33 CG: mean age 50.18 Female 100%	IG: standard care and BREATH CG: standard care 16 weeks The tool	Cancer Empower-ment Question-naire	A statistically non-significant effect on patient empowerment A statistically significant effect on self-efficacy (mean 21.13, SD 0.29, mean difference 0.997, 95% CI 0.21 to 1.78, *p* < 0.05) with small effect size (*d* = 0.28)	10/13
van der Hout et al. 2020 Netherlands [54]	To assess the feasibility and effect of a web-based self-management support intervention with an integrated eHealth application, Oncokompas	RCT	Cancer survivors (*n* = 625) IG: median age 65 49% Female 51% Male CG: median age 65 Female 52% Male 48%	Oncokompas 1–2 weeks The tool	PAM The General Self-Efficacy scale	A statistically non-significant effect on patient activation A statistically non-significant effect on self-efficacy	10/13
Visser et al. 2018 Netherlands [55]	To assess the effect of a mobile/web-based blended care with group medical consultations and tablet-based online app, My-GMC	RCT	People with breast cancer during follow-up (*n* = 109) IG: mean age 55.8 Female 100% GC: mean age 57.9 Female 100%	IG: group medical consultations My-GMC CG: 1 individual medical visit 3 months Peers, a clinical nurse specialist	the Dutch Empowerment Questionnaire for breast cancer patients	A statistically non-significant effect on empowerment	8/13
Wang et al. 2022 China [56]	To explore the effect of a mobile-based shared decision-making assistant (SDM assistant) on the decision-making of informed patients	Quasi-experimental study	People with liver cancer before treatment (*n* = 180) IG: Mean age 50.0 Female 24.4% Male 75% CG: Mean age 51.7 Female 21.1% Male 78.9%	IG: SDM assistant GC: standard care From the date of admission to the completion of decision-making The tool	An adapted version of DSES	A statistically significant effect on self-efficacy (87.75 ± 6.87, *p* < 0.05)	8/9
Wright et al. 2021 UK [57]	To test the feasibility and effect of a web-based self-management program, help to overcome problems effectively (HOPE)	RCT feasibility study	People with cancer during any treatment stage (*n* = 41) Mean age 54.3 Female 78% Male 22%	IG: HOPE GC: wait list 6 weeks Peers	PAM	A statistically non-significant effect on patient activation	10/13
Breen et al. 2017 Australia [58]	To evaluate the feasibility of a mobile-based ASyMS-H, a chemotherapy side-effect monitoring/management system	Qualitative study	People with blood cancers currently receiving/about to commence chemotherapy treatment (*n* = 17) Average age 48.4 Female 27.8% Male 72.2%	ASyMS-H One chemotherapy cycle Nurses	Usage data Semi-structured interviews	Perceived benefits of using ASyMS-H included: reassurance; empowerment; increased health-awareness/adherence to self-care; promotion of timely clinical intervention and improved recall of side-effects and communication with clinicians/family/friends	9/10
Gustavell et al. 2020 Sweden [59]	To describe patients experiences of a mobile-based Interaktor app for symptom reporting and management	Qualitative study	People with cancer after 6 months of pancreaticoduodenectomy (*n* = 26) Mean age 67 Female 31% Male 69%	Interaktor at least 4 weeks Interviews of experiences with using the app Nurses	Interviews Usage data	Theme “Being seen as a person” reflects patients’ experiences of the support for being personally involved in own care and of care based on ones’ personal needs. Access to self-care advice provided new knowledge on symptoms and their self- management, helped to understand own feelings and to deal with misconceptions	6/8
Lambert et al. 2020 Canada [60]	To report the acceptability of a web-based psychosocial and physical activity self-management program, TEMPO	Qualitative study	People with prostate cancer during treatment and follow-up (*n* = 19) Mean age 64.7 Male 100%	TEMPO 10 weeks The tool	Semi-structured interviews	The learned skills were engaging in physical activity and coping (e.g., how to overcome challenges and manage stress)	6/10
Schuit et al. 2021 Netherlands [61]	To gain insight of patients’ experiences of a web-based self-management support intervention with an integrated eHealth application, Oncokompas	Qualitative study	Head and neck cancer survivors and incurably ill people with cancer (*n* = 22) Mean age 65.5 Female 36% Male 64%	Oncokompas 3 months The tool	Semi-structured interviews	Participants’ self-management strategies to cope with cancer were as follows: staying in control, taking responsibility, staying optimistic, seeking distraction, acknowledging symptoms, and finding acceptance A positive aspect of Oncokompas was enabling patients to self-manage	9/10
Skorstad et al. 2022 Norway [62]	To investigate experiences with nurse-led consultations supported by a mobile-based eHealth technology, LETSGO app	Qualitative study	People treated for uterine, ovarian, cervical, or vulvar cancer who originally participated in LETSGO pilot study (*n* = 12) Median age 51.5 Female 100%	LETSGO app 6–7 months after participating in LETSGO pilot study The tool	Semi-structured interviews	Participants’ perceptions of their ability to recognise symptoms was captured in the theme “feeling of increased self-management”. The tool motivated women to become physically more active. Participants gained new and important insights of their cancer diagnosis	8/10
Viitala et al. 2021 Finland [63]	To examine patients’ experiences of using a mobile-based Noona for reporting of chemotherapy symptoms	Qualitative study	People with incurable cancer during palliative and supportive care (*n* = 20) Median age 54 Female 75% Male 25%	Noona Length NR The tool	Semi-structured interviews	The tool promoted coping among people with incurable cancer: active involvement in care, sense of security, sense of freedom, easier communication with professionals, abreast of the treatment, better symptom management	8/10

*IG*, intervention group; *CG*, control group; *PAM,* Patient Activation Measure; *CBI−B*, the Cancer Behaviour Inventory−Brief version; *CB*, Cancer Behavioural Inventory; *Mini−Mac,* the Mini−Mental Adjustment to Cancer Scale; *CG−PAM*, Cregiver Activation Measure; *CASE−Cancer*, Communication and Attitudinal Self−Efficacy Scale for Cancer; *SUPPH−29,* Strategies Used by People to Promote Health; *GSE,* the General Self−Efficacy scale; *PROs*, patient−reported outcomes; *PROMIS*, patient−reported outcome measurement information, self−efficacy for managing emotions and emotional support; *CCQ,* The Cancer Coping Questionnaire; *DSES,* O’Connor’s 11−Item Decision Self−Efficacy Scale. Abbreviations for data collection instruments see Appendix 2 (supplement). Original copyright holders mentioned in the articles. Intervention refers any activities taken by HCPs to promote the well−being of patients with cancer [64]

### Quality appraisal of the studies

Methodological quality or risk of bias were not used as criteria to exclude studies, but merely to show the validity of the results of the review. Assessment was completed by three independent researchers (CC, DC, SM). Disagreements were resolved through discussion until consensus was reached. Quality appraisal was conducted using JBI checklists according to the research design [24]. For RCTs, a 13-item scale (0–13) was used, with focus on internal validity in terms of study, outcome and results, external validity, and statistical conclusion validity. For qualitative studies, a 10-item scale (0–10) was used, with focus on congruity, representation, and accuracy of results. For other quantitative studies, an 8-item scale for cross-sectional studies and a 9-item scale for quasi-experimental studies were used focusing on design accuracy, statistical analysis validity, and internal validity.

## Results

**Fig. 1:**
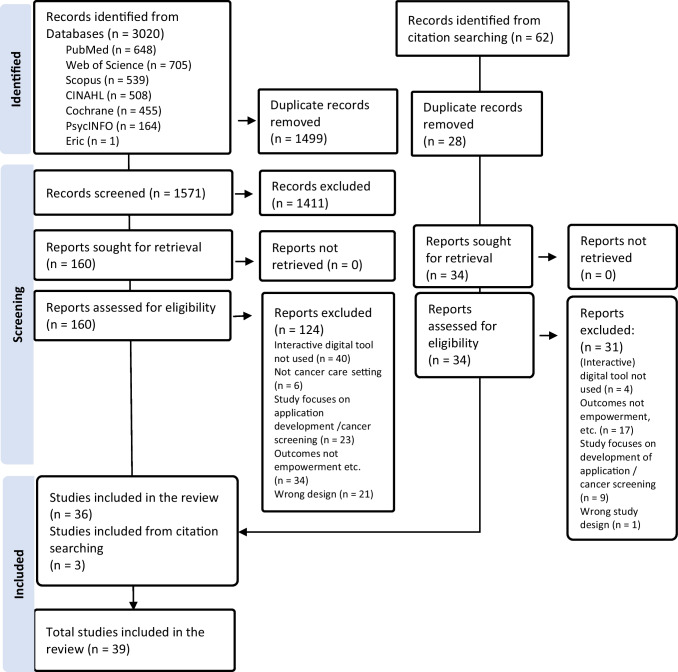
Flowchart of the selection of studies [20]

Of the 3020 records identified from the databases, a total of 36 studies met the inclusion criteria. Three more studies were added based on citation search (Fig. 1).

### Study characteristics

A total of 194 reports were sought for retrieval, and 39 studies were included in the final review: 17 RCTs, 4 of which with feasibility design; 15 single-arm studies with pilot, feasibility, and mixed methods designs; 1 quasi-experimental; and 6 qualitative studies. Most of the studies (25) had been published in the last 5 years, 2018–2022, and the others (14) within 12 years. Most of the studies (15) were Western European: 11 from the Netherlands and 4 from the UK; the others were from Northern Europe (6), the USA (10), Canada (2), Asia (2), and Australia (3). Interaction occurred solely between patients and the IDTs (22) or was attended by peers (7) or HCPs such as nurses (7), physicians (2), psychologists, (2) and social workers (1). In the original studies, the number of interactions was not reported, and their impact on outcomes was not differentiated between HCPs. In accordance with the purpose of the study, only the characteristics of the patient participants are described (Table 2).

### Quality of studies

The Joanna Briggs Institute checklists [24] were used to assess the quality and risk of bias of the studies, see Appendix 3, 4, 5 and 6 (supplement). In randomised control trials (17), all the studies used true randomisation as allocation method and used appropriate statistical analysis. Participants’ characteristics were similar in the comparison groups in almost all studies. Outcomes were measured in the same way for treatment groups in all studies. The design was appropriate in most studies. However, blinding and partial concealment was possible in only one study. Of the quasi-experimental studies (15), all showed appropriate statistical analysis and adequate multiple measurement of outcomes. Participants’ follow-up was completed and clearly described. The research objective was expressed with clarity in all studies. Nonetheless, the studies did not involve comparisons with control groups (CG). All cross-sectional studies (2) showed clear inclusion criteria, setting and objective description, reliable measurement of exposure and outcomes, as well as appropriate statistical analysis design. Nevertheless, only one study included identification of confounding factors, while none had specified strategies relating to this. In qualitative studies (5), congruence of perspective, methods, and objectives was obtained in all studies; similarly, all studies represented accurately the participants’ point of view. Qualitative design was adequate, and data collection was accurately reported in the conclusions. However, only one study identified the researcher’s cultural or theoretical position. There was no recognisable pattern regarding the outcomes of studies with low JBI scores.

### Interactive digital tools supporting patient empowerment

A total of 30 different IDTs were identified. Elements of the IDTs (Table 3) were addressed to patients, home caregivers, or HCPs, but representatives of voluntary sector were not involved. Contact with a HCP, such as a nurse (7), physician (2), psychologist (2), or social worker (1), was involved in a third of the tools. The contents of the elements are detailed in Appendix 7 (supplement).

**Table 3:** Elements and outcomes (outcomes detailed in Table 2) of interactive digital tools

Tools**	Study designs	Elements for patients											Elements for HCPs		Outcomes				
		Symptom monitoring	Self-assessments	Peer support	Information	Action plans	Exercises	Journaling	Quiz	Videos, audio	Tailored information	Alerts to patients	Alerts to HCP	HCP contact to patient	Empowerment	Self-efficacy	Self-management	Coping	Patient activation
ASyMS [40] Mobile-based ASyMS-R [41] Mobile-based ASyMS-H [58] Mobile-based	RCT [40] Single-arm mixed methods [41] Qualitative [58]	x									x		x	x		+ [40] – [41]			
BREATH [53] Web-based	RCT		x		x		x			x	x				–	+			
B-sure [42] Web-based	RCT pilot feasibility				x	x			x							–			
CancerHelp-TT [46] Web-based	RCT				x		x			x						–			
CCO [26, 27] Web-based	Single-arm pilot [26] RCT [27]				x		x	x	x	x								–	
CSSI [47] Mobile-based	Single-arm mixed methods				x		x				x				+				
eRAPID [25] Web-based	RCT	x									x	x				+			–
FOCUS [45] Web-based	Single-arm feasibility		x		x	x					x							–	
Getting down to coping [31] Mobile-based	Single-arm mixed methods			x	x					x						+			
HOPE [57] Web-based	RCT feasibility study		x	x	x	x	x	x	x	x									–
IAYA [48] Mobile-based	Single-arm pilot feasibility			x	x		x									–			
iManage-PC [44] Web-based	Single-arm pilot	x		x	x		x			x	x					+			
Interaktor [59] Mobile-based	Qualitative	x			x								x	x					
LETSGO [62] Web-based	Qualitative	x			x	x	x			x		x							
mPCST-Community [33] Mobile-based	Single-arm pilot	x	x		x					x				x		+			
MyAVL [35] MijnAVL [36] Web-based	Single-arm pilot feasibility [35, 36]		x		x	x	x				x								–
My-GMC [55] Mobile/web-based	RCT			x	x					x				x	–				
Noona [63] Mobile-based	Qualitative	x			x								x						
Oncokompas [32, 43, 50, 54, 61] Web-based	Qualitative [61] RCT [50, 54] Single-arm feasibility [43] Single-arm mixed-methods [32]	x	x								x	x				– [50, 54]			+ [43] – [32, 50, 54]
PatientTIME [52] Web-based	RCT		x		x	x				x	x					+			
PROGRESS [51] Web-based	RCT				x					x						–		+	
SBC [37] Web-based	RCT				x	x				x						+			
SDM Assistant [56] Mobile-based	Quasi-experimental				x		x		x							+			
TEMPO [60] Web-based	Qualitative		x		x	x	x												
The Northwell Head & Neck Health Chats [39] Web-based	Single-arm mixed method	x			x						x			x			–		
The web-based information and support system [30] Web-based	RCT feasibility		x		x						x				–				
TOLF [34] Mobile/web-based	Single-arm pilot	x			x		x				x						+		
True North PN app [28] Web-based	Single-arm pilot feasibility		x	x	x														+
WebChoice [29, 49] Web-based	RCT [29, 49]	x	x	x	x		x	x			x	x		x		–			
WSEDI [38] Web-based	RCT pilot feasibility		x		x	x		x			x					+			

**Full names of the tools in Appendix 2 (supplement); *IDT,* interactive digital tool; +, a statistically significant effect on outcomes; –, a statistically non−significant effect on outcomes

### Elements addressed to patients

IDTs offer patients self-assessments (12) and symptom-monitoring (11). Self-assessments were patient-reported outcomes (PROs) covering physical, social, functional [29, 34, 36, 38, 40, 41, 44, 52, 58, 59], and psychosocial issues [28–30, 45, 49, 52, 53, 57, 60] as well as need for professional help [29, 49]. Symptom-monitoring covered physical, functional, and psychosocial dimensions [25, 34, 40, 41, 44, 49, 58, 63] as well as various quality of life aspects [32, 43, 50, 54, 61].

Tools tailored information (14) to support self-management. Tailored information was triggered by self-assessments, symptom-monitoring, or alerts based on patient records and upcoming appointments [35, 36, 47], self-test results [45, 53], needs’ assessment [52], or by comparing patients’ actual behaviours and recommendations [38]. Tailored exercise was based on PROs or dietary plans [35, 36, 38]. Alerts were triggered by PROs and targeted to HCPs [40, 41, 58, 59, 63] or patients themselves [25, 29, 49, 50, 54, 61, 62].

General information (27), i.e., information not tailored for patients provided cognitive, emotional, and practical support for self-management [25–30, 33, 39, 43–45, 47–49, 52, 55, 56, 58–60, 63], coping with cancer [31, 34, 58], communicating with HCPs [52], and decision-making [26, 27, 42, 56].

Peer-support (7) provided an opportunity to connect and share personal content with other people with cancer [29, 48, 49] facilitated by HCPs [44, 55] or peers [57]. It also helped navigation in the health care system [28].

IDTs offer activities to address commonly experienced physical, emotional, social, and communication difficulties [26] among people with cancer. They were provided in different forms: exercises [26, 27, 29, 34–36, 44, 46–49, 53, 56, 60, 62], action plans [35–38, 52, 57, 60, 62], journaling [26, 27, 29, 38, 47, 57], and quizzes [26, 27, 42, 56, 57].

### Elements addressed to HCPs

The tools had clearly fewer activities addressed to HCPs than to patients. They included the possibility to review PROMs, facilitate chat rooms, or contact patients to provide tailored advice on exceeding reference values by nurses [25, 29, 39, 49, 58, 59], physicians [29, 39], psychologists [31, 33], and social workers [29]. Nurses could also participate in online support group sessions [55] without patients’ self-assessments or initiative.

### Outcomes of the interactive digital tools on patient empowerment and related aspects

The digital tools supporting patients’ empowerment and related aspects had versatile outcomes (Tables 2 and 3). Two kinds of evidence were explored: statistical and experiential. Statistical significance was reached in less than half of the outcomes measuring empowerment and related aspects. These are reported in the text. Patient experiences of empowerment when using IDTs were all positive and are reported at the end of this chapter.

### Empowerment

Four different IDTs were used to explore the effect on patient empowerment as such. Only one single-arm pilot study using the CSSI app to navigate the breast cancer journey [47] was effective. The tool offered mostly information links to reliable webpages and clinical reports. The appropriate content and high quality of the tool had a positive effect on empowerment. The results indicated enhanced sense of control over cancer and general empowerment of women.

### Self-efficacy

Of the 19 studies exploring the effect of IDTs on self-efficacy, ten were effective. Of the 13 RCTs, the studies were e-RAPID on managing side effects during the treatment [25], PatientTIME to support communication among people with lymphoma [52], BREATH to support self-management of people with breast cancer [53], and SBC to manage cancer-related issues [37], the ASyMS to support self-management of chemotherapy-related side effects [40], WSEDI on exercise and intake of fruit and vegetables for people with breast cancer [38], and a quasi-experimental study of an SDM Assistant to support decisions concerning people with liver cancer before the treatment [56]. Three out of five single-arm studies were effective: Getting Down to Coping [31] for people with prostate cancer to support self-management after treatment, mPCST-Community [33] for people with breast cancer to support pain management, and iManage-PC [44] for people with prostate cancer to manage adverse effects during the treatment.

### Coping

A total of four studies assessed the effect of IDTs on patients’ coping. Only one RCT using PROGRESS was effective in redirection of worrying thoughts among people with localised prostate cancer after completion of treatment [51].

### Patient activation

Nine studies assessed the effect of IDTs on patient activation. None of the RCTs were effective. Patient activation was improved in two out of five single-arm studies: the True North PN to support patients’ symptom self-management [28] and the Oncokompas for people with breast cancer [43].

### Self-management

Two single-arm studies assessed the effect of IDTs on patients’ self-management, the TOLF being effective on lymphoedema symptoms among people with breast cancer after surgical treatment [34].

### Patients’ experiences of empowerment and related aspects when using interactive digital tools

There were six qualitative studies exploring patients’ empowering experiences after using IDTs. The overall experience was positive, and two of the tools included interaction with a nurse [58, 59]. The ASyMS-H during chemotherapy increased health-awareness and adherence to self-care among people with blood cancers. [58]. The theme “Being seen as a person” reflected patients’ experiences of support for participation and personal care needs when using the Interaktor [59]. Both tools included symptom monitoring, information, and alerts to HCPs [58, 59]. By using the TEMPO for dyads, patients felt they had gained knowledge and learned coping skills to overcome challenges and manage stress. The tool included self-assessments, information, and exercises [60]. Patients with head and neck cancer perceived that the Oncokompas supported symptom self-management and strategies to cope with cancer, staying in control and taking responsibility for own care. It included symptom monitoring, self-assessments, tailored information, and alerts to the patients [61]. The experience of LETSGO among people with gynaecological cancer was a “feeling of increased self-management”, describing the ability to recognise cancer-related symptoms and motivation to physical exercise. The tool included symptom monitoring, information, exercises, and patient alerts [62]. People with incurable cancer experienced that the Noona enhanced active involvement in care, sense of security and freedom, communication with professionals, being abreast with the treatment, and better symptom management. The tool included information, symptom monitoring, and alerts to nurses [63].

## Discussion

The number of IDTs is growing rapidly with simultaneous research to show its impact on the health outcomes of people with cancer. Digital solutions are becoming more sophisticated, also in supporting patients in their empowerment and recovery. Our purpose was to look at this evidence, focusing on the outcomes of IDTs related to the empowerment of people with cancer.

The IDTs aiming to support patient empowerment are numerous. In our review, we included 39 studies with 30 digital tools that have been developed rather recently in different countries, mostly in Europe or the USA. In all of these, people with cancer are part of the interaction, their role varying from receiving standard information to performing individually tailored activities. This distinction is important from the perspective of empowerment, which assumes that patients are active and have an important role in decision-making and control of their own health [12]. Several activities have been included in the tools such as symptom-monitoring [25, 29, 32, 34, 40, 41, 43, 44, 49, 50, 54, 58, 61, 63], self-assessment of health-related issues [28–30, 34, 36, 38, 40, 41, 44, 45, 49, 52, 53, 57–60], exercises [26, 27, 34, 38, 48, 57, 60], action plans [35–37, 52, 57, 60, 62], journaling [26–29, 38, 49, 57], quizzes [26, 27, 37, 42, 56, 57], and alerts including an opportunity to communicate with HCPs. All these indicate support for empowerment, even though in many studies, a more detailed description of the intensity and implementers of these activities is not clear or may even be lacking, posing a challenge for future researchers and developers. HCPs interacting with patients via IDTs were most often nurses, with other professional groups such as psychologists, physicians, and social workers participating less frequently. We did not see a link between the results and with whom the interaction occurred (patient–HCP, patient–peers, patient–IDT). However, this review does not provide a systematic description about the role of any specific professional group in interactive digital tools.

Unlike previous reviews in the field [13–15, 28], our results introduce both mobile and web-based IDTs supporting patient empowerment. The importance of empowerment has been stated on the level of individual patients [12], professionals [65], and societies [66]. However, empowerment is a multidimensional concept which is difficult to measure with a single instrument [11]. This was the case in our literature search as well: when using the single term “empowerment”, we found a very limited number of studies. Thus, based on the literature, we also used concepts partially expressing the same patient-centred goals as empowerment. We call these “aspects of empowerment” due to their similar nature, but limited scope. This, of course, relates to the results. For example, the concept of self-efficacy was used as part of empowerment and had most of the statistically significant results, but we have to be cautious to conclude that these studies cover the entire concept of empowerment. However, on the other hand, we can conclude that the literature covers patient-oriented IDTs for people with cancer, aiming to support their own activities. Furthermore, the tools support the use of general or tailored information for patients. These are important elements as knowledge is seen as essential for making choices and acting in one’s own interest and thus, being empowered [5]. Statistically significant outcomes of the use of IDTs were identified on empowerment itself [47], self-efficacy [25, 31, 33, 37, 38, 40, 44, 52, 53, 56], coping [55], and patient activation [28, 43]. It should be noted that there were several studies with feasibility design, indicating a need for further testing with larger sample sizes and strict design.

Four IDTs were used in several studies: CCO [26, 27], MyAVL [35, 36], WebChoice [29, 49], and Oncokompas [32, 43, 50, 54, 61]. Of these, only a 1-week single-arm feasibility study using Oncokompas achieved a statistically significant effect on patient activation [43]. We also analysed experiential evidence of the outcomes of IDTs. The experiences of patients were positive [58–63] in terms of improving self-management, increasing knowledge, learning new coping skills, staying in control, and taking responsibility of and participating in own care. These experiences have specific importance when planning interventions and development programmes in clinical practice using these tools. Furthermore, the studies in this review indicate a lot of detailed outcomes of using digital interactive tools, partly in groups of people with specific cancers. These details produce knowledge for those patients as well, even if some of them were investigated in a single study. The duration of the interventions ranged from 1 to 24 weeks, and no association was seen between the length and effectiveness of the intervention. It is notable that large proportion of the studies that achieved significant results (15) were conducted among people with breast cancer [28, 33, 34, 38, 41, 43, 44]. This may be because, based on the CINAHL database, this patient group has been most studied (328 references) compared to people with colorectal cancer (65 references) or prostate cancer (84 references). In addition, compared to men, women use health forums and blogs more often and value their social dimensions, entertainment, as well as the information they offer. In general, the Internet has been a more important source of health information for women than for men [67]. It is important to take gender factors into account when designing digital platforms in order to meet the needs of the target group as well as possible.

In summary, the research on IDTs among people with cancer is promising; this includes tools that have already been tested and those that are still under development. However, many of the RCTs and single-arm trials used feasibility or pilot design. Thus, there is a need for future testing of the tools with larger as well as multinational samples, including new technology, and ensuring the sustainability of successful activities and interventions. Only five studies [26, 31, 43, 44, 53] reported the effect size, which allowed conclusions to be drawn about the magnitude of the results. In these studies, the effect size was small among people with breast cancer and medium among people with prostate cancer or different types of cancer. This review targeted patients, so knowledge of the HCPs’ interacting is very limited; therefore, no conclusions can be drawn about the contributions of different professional groups to patient empowerment.

There are strengths and limitations in this review. The strengths refer to the search strategy and review process: we used several databases with a systematic process. The Covidence tool allowed rapid, reliable evaluations and researcher collaboration. The limitations in the review are related to the search terms, inclusion criteria, and the quality of the studies. The search terms were selected to provide a broad coverage of evidence about the topic. Therefore, not only the term empowerment but also its aspects together with search terms focusing on digital tools in cancer care were included as search terms. This combination resulted in some overlapping hits with extensive search results. Using the AND operator between the term empowerment and the terms indicating its aspects could have provided a more narrowed down result. The inclusion criteria were strict and corresponded with the review aim. However, studies published in languages other than English and publications from other sources were missed. Most of the studies showed an acceptable quality according to the JBI criteria, but due to heterogeneous design, the level of evidence related to each group of studies is different and meta-analysis was omitted. Therefore, we cannot achieve strong evidence. However, most of the studies are intervention studies, indicating that the goal is to achieve strong research evidence.

### Implications for practice, policy, and research

Implications for practice are related to the high number of IDTs as a positive result, indicating the researchers’ aim to develop new methods to support people with cancer. In IDTs, the elements are reasonable, indicating an understanding of the multidimensionality of cancer care. They have, however, mostly been tested only once in a single study or on a limited basis, which is why further testing is required. A good indication is, for example, that the role of patients is included in the tools even though there is variation in the activity of patients. In the future, more empowerment-supporting activities need to be added to the tools to increase their individuality. Moreover, different professional groups are strongly encouraged to participate in the elements addressed to HCPs to support patient empowerment multi-professionally through IDTs.

The implication for health policy is that the number of people with or recovering from cancer is increasing, which calls for a strong position in health policy for this group of patients, increased digital opportunities to realise their self-care and assessment and for monitoring symptoms and recovery. This review is a start for considering the elements of the policies from the perspective of people with cancer.

The implication for future research is that there is an urgent need to strengthen the multi-methodological approach, especially due to the nature of the concept of empowerment. It is too simplified to assume that empowerment of people with cancer could be analysed by any single design. Moreover, modification of IDTs is needed to support the interaction between patients and HCPs and to measure its outcomes, also from the perspective of HCPs. Finally, there is a need to study the cost-effectiveness of digital services in cancer care.

## Conclusion

A plethora of interactive digital tools have been developed and tested in studies, favouring feasibility and pilot designs. These tools encourage patients to be active and have an important role in decision-making and in taking control of their own health. Tailored information is emphasised as knowledge is seen as essential to be empowered. Both statistical and experiential evidence indicates positive outcomes for patient empowerment through interactive digital tools. The tools need to be further tested to confirm the research evidence. People with cancer may be good partners in the future development of these tools.

### Supplementary Information

Below is the link to the electronic supplementary material.

Supplementary file1 (DOCX 17 KB)

Supplementary file2 (DOCX 49 KB)

Supplementary file3 (DOCX 20 KB)

Supplementary file4 (DOCX 17 KB)

Supplementary file5 (DOCX 16 KB)

Supplementary file6 (DOCX 19 KB)

Supplementary file7 (DOCX 61 KB)

## Data Availability

The data that support the findings of this review are available on request from the corresponding author (LT).
