# Correction to: Interactive digital tools to support empowerment of people with cancer: a systematic literature review

**DOI:** 10.1007/s00520-024-08678-x

**Published:** 2024-07-11

**Authors:** Leena Tuominen, Helena Leino‑Kilpi, Jenna Poraharju, Daniela Cabutto, Carme Carrion, Leeni Lehtiö, Sónia Moretó, Minna Stolt, Virpi Sulosaari, Heli Virtanen

**Affiliations:** 1 Department of Nursing Science, University of Turku, Turku, Finland; 2 University of Turku FI and Wellbeing Services County of Southwest Finland, University of Turku FI, Turku University Hospital, Turku, Finland; 3 eHealth Lab Research Group, School of Health Sciences and eHealth Center, Universitat Oberta de Catalunya, Barcelona, Spain; 4 eHealth Lab Research Group, Faculty of Health Sciences Studies, E-Health Center, School of Health Sciences and eHealth Center, Universitat Oberta de Catalunya, Barcelona, Spain; 5 Turku University Library, University of Turku, Turku, Finland; 6 Wellbeing Services County of Satakunta, Pori, Finland; 7 Health and Well-Being, Turku University of Applied Sciences, Turku, Finland; 8 Research Advancing Supportive Cancer and Palliative Care (CARE) - Research Group, Turku, Finland; 9 European Oncology Nursing Society, Brussels, Belgium

Correction to: Supportive Care in Cancer (2024) 32:396.

10.1007/s00520-024-08545-9

The correct name of the third author should be Jenna Poraharju and not Jeena Poraharju.

The below should be the correct list of authors:

Leena Tuominen · Helena Leino‑Kilpi · Jenna Poraharju · Daniela Cabutto · Carme Carrion · Leeni Lehtiö · Sónia Moretó · Minna Stolt · Virpi Sulosaari · Heli Virtanen.

The Publisher regrets this error.

The original article has been corrected.

