# Supplementary material for: Interactive digital tools to support empowerment of people with cancer: a systematic literature review
Source: Support Care Cancer. 2024 May 31;32(6):396. doi: 10.1007/s00520-024-08545-9 (PMC11139693; doi:10.1007/s00520-024-08545-9)
Supplement: Supplementary file 1 — Supplementary file1 (DOCX 17 KB) [file 520_2024_8545_MOESM1_ESM.docx]

**Appendix 1** Literature search

| Database | Search strategy | Filters | Number of results |
| --- | --- | --- | --- |
| Pubmed | ("Empowerment"[Mesh] OR "Self-Management"[Mesh] OR "self-car*" OR "self-manag*" OR "Self-Care"[Mesh] OR empower*[tw] OR coping*[tw] OR "perceived control*"[tw] OR activation*[tw] OR action*[tw] OR "self-efficac*"[tw]) AND (cancer*[tw] OR oncolog*[tw]) AND (digital*[tw] OR digitalisation*[tw] OR digitalization*[tw] OR "e-health*"[tw] OR ehealth*[tw] OR mhealth*[tw] OR "Electronic health*"[tw] OR Telecare*[tw] OR "mobile health*"[tw] OR digitisation*[tw] OR digitization*[tw] OR telecommunication*[tw] OR "Telecommunications"[Mesh] OR "mobile-based*"[tw] OR "tele-based*"[tw] OR "web-based*"[tw] OR "information technolog*"[tw] OR "Information Technology"[Mesh] OR "m-health*"[tw] OR "Digital Technology"[Mesh] OR "Telemedicine"[Mesh] OR telemedicine*[tw] OR telehealth*[tw]) AND ("Patients"[Mesh] OR patient*[tw]) AND (interact*[tw] OR communicat*[tw] OR relat*[tw] OR participator*[tw] OR collaborative*[tw] OR rapport*[tw] OR responsive*[tw] OR multimodal*[tw] OR "remote support*"[tw] OR connect*[tw]) NOT ("Congress"[Publication Type] OR "Review" [Publication Type] OR "Systematic Review"[Publication type] OR "Meta-analysis"[Publication type] OR "Letter" [Publication Type] OR "Editorial" [Publication Type]) NOT (Child*[tw] OR adolesc*[tw]) | English, Published since 2010 | 648 |
| Cinahl | (MH "Empowerment" OR MH "Self-Care+" OR MH "Self-Management" OR MH "Self-Efficacy" OR "self-car*" OR "self-manag*" OR empower* OR coping* OR "perceived control*" OR activation* OR action* OR "self-efficac*") AND (cancer* OR oncolog*) AND (MH "Information Technology+" OR MH "Digital Technology+" OR MH "Telecommunications+" OR MH "Telemedicine+" OR MH "Telehealth+" OR MH "Digital Health+" OR digital* OR digitalisation* OR digitalization* OR "e-health*" OR ehealth* OR mhealth* OR "Electronic health*" OR Telecare* OR "mobile health*" OR digitisation* OR digitization* OR telecommunication* OR "mobile-based*" OR "tele-based*" OR "web-based*" OR "information technolog*" OR "m-health*" OR telemedicine* OR telehealth*) AND (patient* OR MH "Patients+") AND (interact* OR communicat* OR relat* OR participator* OR collaborative* OR rapport* OR responsive* OR multimodal* OR "remote support*" OR connect*) NOT (PT review* OR PT "meta-analysis" OR PT “meta-analysis” OR PT "systematic review") NOT (child* OR adolesc*) | Peer review, English, academic journal, Published since 2010 | 508 |
| Web of Science Core Collection | Topic: (empower* OR "self-car*" OR "self-manag*" OR empower* OR coping* OR "perceived control*" OR activation* OR action* OR "self-efficac*") AND (cancer* OR oncolog*) AND (digital* OR digitalisation* OR digitalization* OR "e-health*" OR ehealth* OR mhealth* OR "Electronic health*" OR Telecare* OR "mobile health*" OR digitisation* OR digitization* OR telecommunication* OR "mobile-based*" OR "tele-based*" OR "web-based*" OR "information technolog*" OR "m-health*" OR telemedicine* OR telehealth*) AND patient* AND (interact* OR communicat* OR relat* OR participator* OR collaborative* OR rapport* OR responsive* OR multimodal* OR "remote support*" OR connect*) NOT (child* OR adolesc*) | Article, early access, English, Published since 2010 | 705 |
| Scopus | (TITLE-ABS(empower* OR "self-car*" OR "self-manag*" OR coping* OR "perceived control*" OR activation* OR action* OR "self-efficac*") OR AUTHKEY("self-car*" OR "self-manag*" OR empower* OR coping* OR "perceived control*" OR activation* OR action* OR "self-efficac*")) AND (TITLE-ABS(cancer* OR oncolog*) OR AUTHKEY (cancer* OR oncolog*)) AND (TITLE-ABS(digital* OR digitalisation* OR digitalization* OR "e-health*" OR ehealth* OR mhealth* OR "Electronic health*" OR Telecare* OR "mobile health*" OR digitisation* OR digitization* OR telecommunication* OR "mobile-based*"OR "tele-based*" OR "web-based*" OR "information technolog*" OR "m-health*" OR telemedicine* OR telehealth*) OR AUTHKEY(digital* OR digitalisation* OR digitalization* OR "e-health*" OR ehealth* OR mhealth* OR "Electronic health*" OR Telecare* OR "mobile health*" OR digitisation* OR digitization* OR telecommunication* OR "mobile-based*" OR "tele-based*" OR "web-based*" OR "information technolog*" OR "m-health*" OR telemedicine* OR telehealth*)) AND (TITLE-ABS(patient*) OR AUTHKEY(patient*)) AND (TITLE-ABS(interact* OR communicat* OR relat* OR participator* OR collaborative* OR rapport* OR responsive* OR multimodal* OR "remote support*" OR connect*) OR AUTHKEY(interact* OR communicat* OR relat* OR participator* OR collaborative* OR rapport* OR responsive* OR multimodal* OR "remote support*" OR connect*)) AND NOT (TITLE-ABS(child* OR adolesc*) OR AUTHKEY(child* OR adolesc*)) | Articles, erratum, English, Published since 2010 | 539 |
| Cochrane | (empower* OR "self-care" OR "self-management" OR "self-caring" OR "self-managing" OR coping* OR perceived NEXT control* OR activation* OR action* OR "self-efficacy") AND (cancer* OR oncolog*) AND (digital* OR digitalisation* OR digitalization* OR "e-health" OR ehealth* OR mhealth* OR Electronic NEXT health* OR Telecare* OR mobile NEXT health* OR digitisation* OR digitization* OR telecommunication* OR "mobile-based" OR "tele-based" OR "web-based" OR information NEXT technolog* OR "m-health" OR telemedicine* OR telehealth*) AND patient* AND (interact* OR communicat* OR relat* OR participator* OR collaborative* OR rapport* OR responsive* OR multimodal* OR remote NEXT support* OR connect*) NOT (child* OR adolesc*) | Only trials, English, Published since 2010 | 455 |
| PsycINFO | (DE "Empowerment" OR DE "Self-Care" OR DE "Self-Management" OR DE "Self-Instructional Training" OR DE "Self-Efficacy" OR empower* OR "self-car*" OR "self-manag*" OR coping* OR "perceived control*" OR activation* OR action* OR "self-efficac*") AND (cancer* OR oncolog*) AND (digital* OR digitalisation* OR digitalization* OR "e-health*" OR ehealth* OR mhealth* OR "Electronic health*" OR Telecare* OR "mobile health*" OR digitisation* OR digitization* OR telecommunication* OR "mobile-based*" OR "tele-based*" OR "web-based*" OR "information technolog*" OR "m-health*" OR telemedicine* OR telehealth*) AND (patient* OR DE "Patients" OR DE "Geriatric Patients" OR DE "Hospitalized Patients" OR DE "Medical Patients" OR DE "Outpatients" OR DE "Patient Safety" OR DE "Psychiatric Patients" OR DE "Surgical Patients" OR DE "Terminally Ill Patients") AND (interact* OR communicat* OR relat* OR participator* OR collaborative* OR rapport* OR responsive* OR multimodal* OR "remote support*" OR connect*) NOT (PZ letter OR PZ review OR PZ editorial OR MR "Systematic review" OR MR "Meta Analysis" OR MR "Literature Review") NOT (child* OR adolesc*) | Peer review, English, Published since 2010 | 164 |
| Eric | (empower* OR "self-car*" OR "self-manag*" OR coping* OR "perceived control*" OR activation* OR action* OR "self-efficac*") AND (cancer* OR oncolog*) AND (digital* OR digitalisation* OR digitalization* OR "e-health*" OR ehealth* OR mhealth* OR "Electronic health*" OR Telecare* OR "mobile health*" OR digitisation* OR digitization* OR telecommunication* OR "mobile-based*" OR "tele-based*" OR "web-based*" OR "information technolog*" OR "m-health*" OR telemedicine* OR telehealth*OR DE "Information Technology" OR DE "Web 2.0 Technologies") AND (patient* OR DE "Patients" OR DE "Hospitalized Children") AND (interact* OR communicat* OR relat* OR participator* OR collaborative* OR rapport* OR responsive* OR multimodal* OR "remote support*" OR connect*) NOT (child* OR adolesc*) | Peer review, English, Published since 2010 | 1 |

Interactive digital tools to support empowerment of people with cancer: a systematic literature review Supportive Care in Cancer

Corresponding author:

Leena Tuominen*****

University of Turku

Department of Nursing Science

20014 University of Turku, Finland

[leetuo@utu.fi](mailto:leetuo@utu.fi)

Authors:

Leino-Kilpi Helena*****

Poraharju Jenna

Cabutto Daniela

Carrion Carme

Lehtiö Leeni

Moretó Sònia

Stolt Minna

Sulosaari Virpi

Virtanen Heli

* Shared position of first author
