# Supplementary material for: Interactive digital tools to support empowerment of people with cancer: a systematic literature review
Source: Support Care Cancer. 2024 May 31;32(6):396. doi: 10.1007/s00520-024-08545-9 (PMC11139693; doi:10.1007/s00520-024-08545-9)
Supplement: Supplementary file 2 — Supplementary file2 (DOCX 49 KB) [file 520_2024_8545_MOESM2_ESM.docx]

**Appendix 2** Abbreviations and full names of the digital tools

| Abbreviation | Full Name |
| --- | --- |
| ASyMS[40] | The Advanced Symptom Management System |
| ASyMS-H[58] | The Advanced Symptom Management System for Hematology |
| ASYMS-R[41] | The Advanced Symptom Management System for Radiotherapy |
| BREATH[53] | The Breast Cancer E-Health |
| B-Sure[42] | Not defined |
| CancerHelp-TT[46] | CancerHelp-Talking Touchscreen |
| CCO[26, 27] | Cancer Coping Online |
| CSSI app[47] | Centro di Senologia della Svizzera Italiana |
| eRAPID[25] | The electronic patient self-reporting of adverse-events: patient information and advice |
| FOCUS Program[45] | Not defined |
| Getting Down to Coping[31] | Not defined |
| HOPE[57] | Help to Overcome Problems Effectively |
| IAYA[48] | Not defined |
| iManage-PC[44] | Not defined, PC= Prostate cancer |
| Interaktor app[59] | Not defined |
| LETSGO app[62] | Not defined |
| MijnAVL[36] | AVL: Antoni van Leeuwenhoek hospital |
| MyAVL[35] | MijnAVL in English |
| mPCST-Community[33] | the mHealth Pain Coping Skills Training |
| My-GMC[55] | My Group Medical Consultations |
| Noona[63] | Not defined |
| Oncokompas[32, 43, 50, 54, 61] | Not defined |
| PatientTIME[52] | Not defined |
| PROGRESS[51] | Prostate Cancer Online Guide & Resource for Electronic Survivorship |
| SBC[37] | Springboard Beyond Cancer |
| SDM assistant[56] | Shared Decision-Making Assistant |
| TEMPO[60] | Tailored, web-based, psychosocial and physical activity self-management program |
| The Northwell Head & Neck Health Chats[39] | Not defined |
| The web-based information and support system[30] | Not defined |
| TOLF[34] | The-Optimal-Lymph-Flow |
| True North PN[28] | True North Peer Navigation |
| WebChoice[29, 49] | Not defined |
| WSEDI[38] | A Web-based Self-management Exercise and Diet Intervention |

Interactive digital tools to support empowerment of people with cancer: a systematic literature review Supportive Care in Cancer

Corresponding author:

Leena Tuominen*, University of Turku, Department of Nursing Science, 20014 University of Turku, Finland

[leetuo@utu.fi](mailto:leetuo@utu.fi)

Authors: Leino-Kilpi Helena*, Poraharju Jenna, Cabutto Daniela, Carrion Carme, Lehtiö Leeni, Moretó Sònia, Stolt Minna, Sulosaari Virpi, Virtanen Heli

* Shared position of first author
