# Supplementary material for: Interactive digital tools to support empowerment of people with cancer: a systematic literature review
Source: Support Care Cancer. 2024 May 31;32(6):396. doi: 10.1007/s00520-024-08545-9 (PMC11139693; doi:10.1007/s00520-024-08545-9)
Supplement: Supplementary file 3 — Supplementary file3 (DOCX 20 KB) [file 520_2024_8545_MOESM3_ESM.docx]

**Appendix 3** Methodological quality of included RCTs

| Article | 1 | 2 | 3 | 4 | 5 | 6 | 7 | 8 | 9 | 10 | 11 | 12 | 13 | Overall |
| --- | --- | --- | --- | --- | --- | --- | --- | --- | --- | --- | --- | --- | --- | --- |
| Absolom et al. 2021 | Y | Y | Y | N | N | Y | U | Y | Y | Y | Y | Y | Y | 10/13 |
| Beatty et al. 2016 | Y | Y | Y | N | U | Y | U | Y | Y | Y | Y | Y | Y | 10/13 |
| Bouma et al. 2017 | Y | Y | Y | N | N | Y | N | Y | Y | Y | Y | Y | Y | 10/13 |
| Børøsund, et al. 2014 | Y | Y | Y | N | U | Y | U | Y | Y | Y | Y | Y | Y | 10/13 |
| Leach et al. 2022 | Y | U | Y | U | U | Y | U | Y | Y | Y | Y | Y | Y | 9/13 |
| Lee et al. 2014 | Y | Y | Y | Y | U | Y | U | Y | Y | Y | Y | Y | Y | 11/13 |
| Maguire et al. 2021 | Y | Y | Y | N | U | Y | Y | Y | Y | Y | Y | Y | Y | 11/13 |
| Manne et al. 2020 | Y | Y | U | N | N | Y | U | Y | Y | Y | Y | Y | U | 8/13 |
| Peipert et al. 2021 | Y | Y | Y | N | N | Y | U | Y | Y | Y | Y | Y | U | 9/13 |
| Ruland et al. 2013 | Y | Y | Y | N | U | Y | U | Y | Y | Y | Y | Y | Y | 10/13 |
| Schuit et al. 2022 | Y | N | Y | N | N | Y | N | Y | Y | Y | Y | Y | Y | 9/13 |
| Tagai et al. 2021 | Y | U | Y | U | N | Y | U | Y | Y | Y | Y | Y | Y | 9/13 |
| Van Bruinessen et al. 2016 | Y | U | Y | N | U | Y | N | Y | Y | Y | Y | Y | Y | 9/13 |
| Van den Berg et al. 2015 | Y | Y | Y | N | N | Y | U | Y | Y | Y | Y | Y | Y | 10/13 |
| Van Der Hout et al. 2020 | Y | Y | Y | N | N | Y | N | Y | Y | Y | Y | Y | Y | 10/13 |
| Visser et al. 2018 | Y | N | Y | N | N | U | N | Y | Y | Y | Y | Y | Y | 8/13 |
| Wright et al. 2021 | Y | Y | Y | N | N | Y | U | Y | Y | Y | Y | Y | Y | 10/13 |

*_Y,_* _Yes;_ *_N,_* _No;_ *_U,_* _Unclear._

_1. Was true randomization used for assignment of participants to treatment groups? 2. Was allocation to treatment groups concealed? 3. Were treatment groups similar at the baseline? 4. Were participants blind to treatment assignment? 5. Were those delivering the treatment blind to treatment assignment? 6. Were treatment groups treated identically other than the intervention of interest? 7. Were outcome assessors blind to treatment assignment? 8. Were outcomes measured in the same way for treatment groups? 9. Were outcomes measured in a reliable way? 10. Was follow up complete and if not, were differences between groups in terms of their follow up adequately described and analysed? 11. Were participants analysed in the groups to which they were randomized? 12. Was appropriate statistical analysis used? 13. Was the trial design appropriate and any deviations from the standard RCT design accounted for in the conduct and analysis of the trial?_

Interactive digital tools to support empowerment of people with cancer: a systematic literature review Supportive Care in Cancer

Corresponding author:

Leena Tuominen*

University of Turku

Department of Nursing Science

20014 University of Turku, Finland

[leetuo@utu.fi](mailto:leetuo@utu.fi)

Authors:

Leino-Kilpi Helena*

Poraharju Jenna

Cabutto Daniela

Carrion Carme

Lehtiö Leeni

Moretó Sònia

Stolt Minna

Sulosaari Virpi

Virtanen Heli

* Shared position of first author
