# Supplementary material for: Interactive digital tools to support empowerment of people with cancer: a systematic literature review
Source: Support Care Cancer. 2024 May 31;32(6):396. doi: 10.1007/s00520-024-08545-9 (PMC11139693; doi:10.1007/s00520-024-08545-9)
Supplement: Supplementary file 4 — Supplementary file4 (DOCX 17 KB) [file 520_2024_8545_MOESM4_ESM.docx]

**Appendix 4** Methodological quality of included qualitative studies [26]

| Article | 1 | 2 | 3 | 4 | 5 | 6 | 7 | 8 | 9 | 10 | Overall |
| --- | --- | --- | --- | --- | --- | --- | --- | --- | --- | --- | --- |
| Breen et al. 2017 | Y | Y | Y | Y | Y | U | Y | Y | Y | Y | 9/10 |
| Lambert et al. 2020 | Y | Y | Y | U | U | N | U | Y | Y | Y | 6/10 |
| Schuit et al 2021 | Y | Y | Y | Y | Y | U | Y | Y | Y | Y | 9/10 |
| Skorstad et al. 2022 | Y | Y | Y | U | U | Y | Y | Y | Y | Y | 8/10 |
| Viitala et al. 2021 | Y | Y | Y | U | Y | U | Y | Y | Y | Y | 8/10 |

*_Y_*_, yes;_ *_N_*_, No;_ *_U_*_, Unclear._

_1. Is there congruity between the stated philosophical perspective and the research methodology? 2. Is there congruity between the research methodology and the research question or objectives? 3. Is there congruity between the research methodology and the methods used to collect data? 4. Is there congruity between the research methodology and the representation and analysis of data? 5. Is there congruity between the research methodology and the interpretation of results? 6. Is there a statement locating the researcher culturally or theoretically? 7. Is the influence of the researcher on the research, and vice- versa, addressed? 8. Are participants, and their voices, adequately represented? 9. Is the research ethical according to current criteria or, for recent studies, and is there evidence of ethical approval by an appropriate body? 10. Do the conclusions drawn in the research report flow from the analysis, or interpretation, of the data?_

Interactive digital tools to support empowerment of people with cancer: a systematic literature review Supportive Care in Cancer

Corresponding author:

Leena Tuominen*

University of Turku

Department of Nursing Science

20014 University of Turku, Finland

[leetuo@utu.fi](mailto:leetuo@utu.fi)

Authors:

Leino-Kilpi Helena*

Poraharju Jenna

Cabutto Daniela

Carrion Carme

Lehtiö Leeni

Moretó Sònia

Stolt Minna

Sulosaari Virpi

Virtanen Heli

* Shared position of first author
