# Supplementary material for: Interactive digital tools to support empowerment of people with cancer: a systematic literature review
Source: Support Care Cancer. 2024 May 31;32(6):396. doi: 10.1007/s00520-024-08545-9 (PMC11139693; doi:10.1007/s00520-024-08545-9)
Supplement: Supplementary file 5 — Supplementary file5 (DOCX 16 KB) [file 520_2024_8545_MOESM5_ESM.docx]

**Appendix 5** Methodological quality of included cross sectional studies [27]

| Article | 1 | 2 | 3 | 4 | 5 | 6 | 7 | 8 | Overall |
| --- | --- | --- | --- | --- | --- | --- | --- | --- | --- |
| Gustavell et al. 2020 | Y | Y | Y | Y | U | U | Y | Y | 6/8 |
| Petrocchi et al. 2021 | Y | Y | Y | Y | Y | U | Y | Y | 7/8 |

*_Y_*_, yes;_ *_N_*_, No;_ *_U_*_, Unclear._

_1. Were the criteria for inclusion in the sample clearly defined? 2. Were the study subjects and the setting described in detail? 3. Was the exposure measured in a valid and reliable way? 4. Were objective, standard criteria used for measurement of the condition? 5. Were confounding factors identified? 6. Were strategies to deal with confounding factors stated? 7. Were the outcomes measured in a valid and reliable way? 8. Was appropriate statistical analysis used?_

Interactive digital tools to support empowerment of people with cancer: a systematic literature review

Supportive Care in Cancer

Corresponding author:

Leena Tuominen*

University of Turku

Department of Nursing Science

20014 University of Turku, Finland

[leetuo@utu.fi](mailto:leetuo@utu.fi)

Authors:

Leino-Kilpi Helena*

Poraharju Jenna

Cabutto Daniela

Carrion Carme

Lehtiö Leeni

Moretó Sònia

Stolt Minna

Sulosaari Virpi

Virtanen Heli

* Shared position of first author
