# Supplementary material for: Interactive digital tools to support empowerment of people with cancer: a systematic literature review
Source: Support Care Cancer. 2024 May 31;32(6):396. doi: 10.1007/s00520-024-08545-9 (PMC11139693; doi:10.1007/s00520-024-08545-9)
Supplement: Supplementary file 6 — Supplementary file6 (DOCX 19 KB) [file 520_2024_8545_MOESM6_ESM.docx]

**Appendix 6** Methodological quality of included quasi-experimental studies [28]

| Article | 1 | 2 | 3 | 4 | 5 | 6 | 7 | 8 | 9 | Overall |
| --- | --- | --- | --- | --- | --- | --- | --- | --- | --- | --- |
| Beatty et al. 2011 | Y | Y | NA | N | Y | Y | NA | Y | Y | 6/9 |
| Bender et al. 2022 | Y | Y | U | N | Y | Y | NA | Y | Y | 6/9 |
| Cockle-Hearne et al. 2018 | Y | Y | NA | N | Y | Y | Y | Y | Y | 7/9 |
| De Veer et al. 2020 | Y | Y | NA | N | Y | Y | NA | Y | Y | 6/9 |
| Dorfman et al. 2019 | Y | Y | NA | N | Y | Y | NA | Y | Y | 6/9 |
| Fu et al. 2016 | Y | Y | U | N | Y | Y | NA | Y | Y | 6/9 |
| Groen et al. 2017 | Y | Y | NA | N | Y | Y | NA | Y | Y | 6/9 |
| Kuijpers et al. 2016 | Y | Y | NA | N | Y | Y | NA | Y | Y | 6/9 |
| Ma et al. 2021 | Y | Y | U | N | Y | Y | U | Y | Y | 6/9 |
| Maguire et al. 2015 | Y | Y | NA | N | Y | Y | NA | Y | Y | 6/9 |
| Melissant et al. 2018 | Y | Y | NA | N | Y | Y | NA | Y | Y | 6/9 |
| Murphy et al. 2022 | Y | Y | NA | N | Y | Y | NA | Y | Y | 6/9 |
| Northouse et al. 2014 | Y | Y | NA | N | Y | Y | NA | Y | Y | 6/9 |
| Poort et al. 2021 | Y | Y | U | N | Y | Y | NA | Y | Y | 6/9 |
| Wang et al. 2022 | Y | Y | U | Y | Y | Y | Y | Y | Y | 8/9 |

*_Y_*_, yes;_ *_N_*_, No;_ *_U_*_, Unclear;_ *_N/A_*_, not applicable._

_1. Is it clear in the study what is the cause and what is the effect (i.e., there is no confusion about which variable comes first)? 2. Were the participants included in any comparisons similar? [NOTE: In one single group pre-test/post-test studies where the patients are the same (the same one group) in any pre-post comparisons, the answer to this question should be ‘yes.’] 3. Were the participants included in any comparisons receiving similar treatment/care, other than the exposure or intervention of interest? 4. Was there a control group? 5. Were there multiple measurements of the outcome both pre and post the intervention/exposure? 6. Was follow up complete and if not, were differences between groups in terms of their follow up adequately described and analysed? 7. Were the outcomes of participants included in any comparisons measured in the same way? 8. Were outcomes measured in a reliable way? 9. Was appropriate statistical analysis used?_

Interactive digital tools to support empowerment of people with cancer: a systematic literature review Supportive Care in Cancer

Corresponding author:

Leena Tuominen*

University of Turku

Department of Nursing Science

20014 University of Turku, Finland

[leetuo@utu.fi](mailto:leetuo@utu.fi)

Authors:

Leino-Kilpi Helena*

Poraharju Jenna

Cabutto Daniela

Carrion Carme

Lehtiö Leeni

Moretó Sònia

Stolt Minna

Sulosaari Virpi

Virtanen Heli

* Shared position of first author
