# Supplementary material for: Interactive digital tools to support empowerment of people with cancer: a systematic literature review
Source: Support Care Cancer. 2024 May 31;32(6):396. doi: 10.1007/s00520-024-08545-9 (PMC11139693; doi:10.1007/s00520-024-08545-9)
Supplement: Supplementary file 7 — Supplementary file7 (DOCX 61 KB) [file 520_2024_8545_MOESM7_ESM.docx]

**Appendix 7** Content of the elements of interactive digital tools

|  | Contents for patients | | | | | | | | | Content for patients and HCPs |
| --- | --- | --- | --- | --- | --- | --- | --- | --- | --- | --- |
| Study  Tools | Symptom monitoring | Self-assessments | Tailored information | Information | Peer support | Action plans  Exercises | Journaling | Quiz | Videos  Audios | Alerts |
| eRAPID  [25] | Weekly online symptom reporting |  | Symptom self-management strategies.  Immediate  severity-dependent advice on symptom management or a prompt to contact the hospital. | Feedback and advice to support symptom self-management |  |  |  |  |  | Alerts for severe symptom  reports were sent to each clinical team,  monitored by nurses. |
| CCO [26, 27] |  |  |  | Starting  treatment, coping with physical symptoms,  side effects, and emotional distress, completing treatment |  | Relaxation and meditation exercises. Addressing symptoms such as fatigue, pain, and insomnia, activity and psychosexual needs, communication and needs’ assessment worksheets. | Personal blog for online-journal writing, psychosexual worksheets, therapeutic writing activities, cognitive restructuring diaries to help patients to cope with emotional distress, depressionanxiety, and anger | Immediate-feedback quizzes | Guided relaxation, meditation, exercises mind-fulness, audio-tracks |  |
| True North PN app [28] |  | Assessment of psychosocial issues, preferences for peer navigator, needs and barriers to care |  | Provides practical, informational  and emotional support | Offered peer support for people with prostate cancer as they navigate health and social service systems |  |  |  |  |  |
| WebChoice  [29, 49] | Monitor  the symptoms, problems, and priorities for support along  physical, functional, and psychosocial dimensions. | Physical, social, psychosocial and functional issues, need for professional help | Symptom self-management strategies and  support | Access to  other reliable web sources | Online forum  group discussion facilitated by HCPs, sharing experiences with  other patients via private messages |  | An electronic diary where patients could make personal  notes. |  |  | The patient questions were  asynchronous and were answered within 2 work days by nurses, physicians or social workers. |
| Web-based information and support system [30] |  | Self-screening  of care needs, physical and psychosocial problems | Automated personalised feedback  upon the reported distress score, referral to HCPs | General information about cancer and diagnostic procedures,  treatments, and their side effects |  |  |  |  |  | HCPs (professionals not reported in detail). |
| Getting Down to Coping [31] |  |  |  | Links to medical,  physical, emotional, social, and financial prostate cancer  information | Theory-driven peer support films and a platform for interactive support via an  asynchronous chat forum, facilitated by HCPs. |  |  |  |  | Psychological practitioners and nurses facilitated the chat  forum. |
| Oncokompas  [32, 43, 50, 54, 61] | Monitors various QOL aspects by means  of patient-reported outcome measures |  | Symptom self-management strategies including tips, brochures, and links  to relevant websites and supportive care services. Automatically generated tailored feedback  A selection of personal relevant  topics. | Physical, psychological, social domains, healthy lifestyle, existential issues, breast cancer |  |  |  |  |  |  |
| mPCST-Community [33] |  | Daily  pain ratings | Tailored text messages based on pain ratings and skill practice experiences | Stories about pain experiences  from example participants, and other materials |  |  |  |  | Video-clips modelling coping skills, guided relaxation and meditation exercises | Daily pain ratings and skills practice experience were sent to psychologists who promoted self-efficacy via tailored text messages. |
| TOLF  [34] | Monitor the  lymphedema symptoms | Assess the lymphedema symptoms to determine the frequency of lymphatic and limb mobility exercises | Lymphedema and symptom self-management strategies, overview of past and upcoming  appointments, |  |  | Physical activity.  Lymphatic and limb mobility exercises. |  |  | Learn daily lymphatic exercises by watching and following the avatar simulation videos |  |
| MyAVL  [35]  MijnAVL  [36] |  | Quality of life | Personalised patient education material of disease, treatment, and side effects, tailored physical activity  advice, PROs and related feedback | Access to the patient records. |  | Physical activity, setting appropriate goals and making an action plan related to physical activity. |  |  |  |  |
| SBC  [37] |  |  |  |  |  | Patients were supported to develop a tailored action plan to manage their own cancer-related issues. |  |  |  |  |
| WSEDI  [38] |  | Physical, social and functional issues | Exercise and dietary behaviour, daily automatic feedback on goal  achievement |  |  | Setting appropriate goals and plan exercise behavior and diet. | Diaries focused on daily activity and diet |  |  |  |
| The Northwell Head & Neck Health Chats [39] | Reporting adverse events |  | Customised easy-to-understand educational material regarding any  symptoms reported | A comprehensive list of self-care education topics  was available |  | Weekly scheduled chats. |  |  |  | Nurses,  advanced care practitioners, and physicians could review reports for all generated patient responses. |
| ASyMS [40] | Monitoring chemotherapy toxicity (10 symptoms) | Assessing chemotherapy toxicity (10 symptoms) | Evidence-based symptom self-care advice |  |  |  |  |  |  | Alerts from mild to moderate symptoms and chemotherapy emergencies. HCPs contact the patient (professionals not reported in detail). |
| ASyMS-R  [41] | monitoring symptoms in people with lung cancer receiving radiotherapy |  | Symptom self-management strategies |  |  |  |  |  |  | Alerts from mild to moderate symptoms and chemotherapy emergencies. HCPs contact the patient (professionals not reported in detail). |
| B-sure  [42] |  |  |  | Information of contralateral prophylactic mastectomy |  | Making a plan to reduce the risk of breast cancer recurrence. |  | Knowledge tests of different treatment options |  |  |
| iManage-PC  [44] | Urinary, bowel, sexual and emotional functioning in people with prostate cancer | Weekly symptom assessments about urinary, bowel, sexual and emotional  functioning. | Symptom self-management strategies | Resource library, a community board to  ask questions and leave comments, monitored by study team. | Monitored by HCPs. | Encouraging patients to learn and practice to manage physiological and psychological symptoms. |  |  | A brief advice on self-management of anxiety as well as sexual, urinary, and bowel adverse effects |  |
| FOCUS Program  [45] |  | Psychosocial issues | Tailored messages to enhance mutual support among dyads | Information related to family and cancer |  | Promoting dyadic interactions. |  |  |  |  |
| CancerHelp-TT  [46] |  |  | An option to generate and print tailored, personally relevant  checklists of issues they may wish to discuss during medical  visits, and select modules that were most important to oneself |  |  | Creating personally relevant checklists of issues to discuss during medical visits. |  |  | Video introduction by a clinician from each site |  |
| CSSI app  [47] |  |  |  | Access  reliable information related to breast cancer and the treatment  process, links to reliable breast cancer websites. |  |  |  |  |  |  |
| IAYA  [48] |  |  |  | A combination of  psychoeducational resources, coping skills training | Opportunity to connect and share personal content with peers independently in a group | Coping exercises to help treat people with anxiety and  mood difficulties. |  |  |  |  |
| PROGRESS  [51] |  |  |  | Written  information, tools to help  participants identify values, goals, and support complementary behaviors |  | Identifying goals, values, and supporting complementary behaviors. |  |  | 58 short videos (47-180 seconds) of simulated patients  demonstrating different communication skills during medical  encounters |  |
| PatientTIME  [52] |  | Psychosocial issues, communication with HCP | Personalised messages and  questionnaires before and after clinical consultations |  |  | Patients were supported in setting appropriate goals and making an action plan to stay motivated. Encouragement to set goals and prepare questions before physician appointment. |  |  | Communication skills during medical encounters |  |
| BREATH  [53] |  | Psychosocial issues, 10 self-tests with automated  feedback |  |  |  | Assignments including 48 different tasks  or homework. |  |  | Videos on adjustment to breast cancer with 20 topics |  |
| Group medical consultations My-GMC  [55] |  |  |  | Standard information  concerning breast cancer survivorship. Suggestions for relevant websites | In three online support group sessions patients were invited to address their own themes in the presence of HCP. | Patients were provided specifically programmed tablets  including several existing apps (e.g., eBooks) |  |  | 13 short videos  showed fragments of interviews with three BCS | During the video GMC patients met in the presence of a clinical nurse specialist who was present for questions and, if necessary, to join the discussion. |
| SDM Assistant  [56] |  |  |  | Knowledge about primary liver cancer-related  treatment. Comparing alternative treatment options  on app. Exploring the preferences. |  | Scoring risks and benefits of treatments, thus assisting patients’ choice. |  | Knowledge tests of different treatment options |  |  |
| HOPE  [57] |  | Psychosocial issues |  | Links to videos, podcasts, and  websites. Information of stress management, fatigue, body image and communication, physical activity, fear of recurrence | Facilitated by trained peers | Coping exercises. Setting appropriate goals and making an action plan related to physical activity. Positivity ratio test and positive  and negative emotions test. Goal-setting | Interactive diaries focused on fatigue, pacing and gratitude | Focused on main challenges faced by cancer survivors | Managing stress, setting goals, managing fatigue and self-compassion, guided relaxation and meditation exercises |  |
| ASyMS-H  [58] | Presence/absence of rigors, nausea, vomiting, constipation diarrhoea, mucositis, peripheral neuropathy, bleeding, fatigue and impairments in daily living activities | The severity and the bother caused by the issue. |  | Application presented evidence-based self-care, access a library of self-care information or view historical side-effect graphs. |  |  |  |  |  | When patient-reported data exceeded predetermined thresh-olds an alert was generated via text message to nurses who contacted the patient. |
| Interaktor  [59] |  | Assessment of self-reported symptoms |  | Access to evidence-based self-care advice and links to relevant websites |  |  |  |  |  | The yellow and red alerts trigger self-care advices for the patient and a text message is automatically sent to nurses. |
| TEMPO  [60] |  | Psychosocial issues, challenges with the dyad |  | A health library (45 factsheets) of self-management and physical activity, examples of light, moderate, and hard exercises for dyads. |  | Physical activity, setting appropriate goals and making an action plan related to physical activity |  |  |  |  |
| LETSGO  [62] |  |  |  |  |  | Physical activity, setting appropriate goals and making an action plan related to physical activity |  |  | Videos of disease, helping patients to manage symptoms or be physically active |  |
| Noona  [63] | Reporting side-effects and symptoms of chemotherapy |  | Modules tailored for different types of cancer patients to target the most relevant symptoms during and after the chemotherapy. |  |  |  |  |  |  |  |

Interactive digital tools to support empowerment of people with cancer: a systematic literature review Supportive Care in Cancer

Corresponding author:

Leena Tuominen*

University of Turku

Department of Nursing Science

20014 University of Turku, Finland

[leetuo@utu.fi](mailto:leetuo@utu.fi)

Authors:

Leino-Kilpi Helena*

Poraharju Jenna

Cabutto Daniela

Carrion Carme

Lehtiö Leeni

Moretó Sònia

Stolt Minna

Sulosaari Virpi

Virtanen Heli

* Shared position of first author
